# A systematic review of metabolomic profiling of gastric cancer and esophageal cancer

**DOI:** 10.20892/j.issn.2095-3941.2019.0348

**Published:** 2020-02-15

**Authors:** Sha Huang, Yang Guo, Zhexuan Li, Yang Zhang, Tong Zhou, Weicheng You, Kaifeng Pan, Wenqing Li

**Affiliations:** ^1^Key Laboratory of Carcinogenesis and Translational Research (Ministry of Education/Beijing), Department of Cancer Epidemiology, Peking University Cancer Hospital & Institute, Beijing 100142, China; ^2^Joint International Research Center of Translational and Clinical Research, Beijing 100142, China

**Keywords:** Gastric cancer, esophageal cancer, metabolomics, Warburg effect, biomarkers

## Abstract

**Objective:** Upper gastrointestinal (UGI) cancers, predominantly gastric cancer (GC) and esophageal cancer (EC), are malignant tumor types with high morbidity and mortality rates. Accumulating studies have focused on metabolomic profiling of UGI cancers in recent years. In this systematic review, we have provided a collective summary of previous findings on metabolites and metabolomic profiling associated with GC and EC.

**Methods:** A systematic search of three databases (Embase, PubMed, and Web of Science) for molecular epidemiologic studies on the metabolomic profiles of GC and EC was conducted. The Newcastle–Ottawa Scale (NOS) was used to assess the quality of the included articles.

**Results:** A total of 52 original studies were included for review. A number of metabolites were differentially distributed between GC and EC cases and non-cases, including those involved in glycolysis, anaerobic respiration, tricarboxylic acid cycle, and protein and lipid metabolism. Lactic acid, glucose, citrate, and fumaric acid were among the most frequently reported metabolites of cellular respiration while glutamine, glutamate, and valine were among the most commonly reported amino acids. The lipid metabolites identified previously included saturated and unsaturated free fatty acids, aldehydes, and ketones. However, the key findings across studies to date have been inconsistent, potentially due to limited sample sizes and the majority being hospital-based case-control analyses lacking an independent replication group.

**Conclusions:** Studies on metabolomics have thus far provided insights into etiological factors and biomarkers for UGI cancers, supporting the potential of applying metabolomic profiling in cancer prevention and management efforts.

## Introduction

Upper gastrointestinal (UGI) cancers, predominantly gastric cancer (GC) and esophageal cancer (EC), are major malignancies in China and worldwide^1^, with prognosis remaining poor in many countries without effective screening programs^2,3^. Holistic promotion of etiological research and identification of novel biomarkers is essential to ensure implementation of timely and appropriate preventive and treatment strategies. Developments in molecular biology, along with emergence of various new omics techniques, have provided powerful tools for advancement of molecular epidemiologic studies on UGI cancers.

Metabolic dysregulation has been shown to underlie carcinogenesis of UGI cancers^4^. In addition to the alterations in glucose metabolism, as indicated by the well-known Warburg effect, dysregulated metabolism of amino acids, lipids, and nucleotides has been demonstrated, both *in vitro* and *in vivo*^5–7^. Metabolites represent the end product of complex joint effects of intrinsic metabolism, environmental exposures, and genetic predisposition. High-throughput metabolomics techniques can facilitate comprehensive identification and quantitative profiling of the entire spectrum of endogenous low molecular weight metabolites (< 1000 Da) in a single sample^8,9^, which may not only aid in identifying promising novel biomarkers but also provide insights into cancer etiology, leading to the development of novel preventive approaches and therapeutic targets^10^.

Studies have been conducted to investigate the broad network of metabolites in UGI cancers based on various human biological samples, including tissue, plasma, and urine^11^. Although efforts have been made to review past literature on the metabolomics of UGI cancers^4,11–14^, these reports were simply narrative descriptions. Only one systematic review was available as of 2012, which included 20 references^4^. In view of the accumulating studies on metabolomic profiling of UGI cancers over the last 6 years, an updated systematic review is warranted to summarize the available literature for a clear understanding of the field of metabolomic studies on UGI cancers and identify specific metabolites and metabolic pathways consistently associated with these cancer types.

To address this issue, we conducted a systematic review of the currently available metabolomic studies on GC and EC. Given the described major interests and our long-standing top priority as cancer epidemiologists to promote cancer prevention and management at the population level, we focused on previous human molecular epidemiologic studies on metabolomic profiling of UGI cancers. Here, we present a summary of the latest advances in determining the individual metabolites and metabolic pathways associated with these cancers while highlighting the limitations of the available studies, with the aim of providing insights into future metabolomic approaches, promoting etiologic research and precision prevention and control of UGI cancers.

## Materials and methods

This study was performed and presented following the requirements of the Preferred Reporting Items for Systematic Reviews and Meta-Analyses (PRISMA statement)^15^ and Preferred Reporting Items for Systematic Review and Meta-Analysis protocols (PRISMA-P)^16^.

### Search strategy and data extraction

We searched the literature for studies focusing on metabolomic profiling of human GC and EC as of September 4, 2019, using Embase, PubMed, and Web of Science databases. Multiple combinations of the keywords, including “mass spectrometry/nuclear magnetic resonance spectroscopy”, “metabolomic/metabonomic/metabolic profiling”, “gastric cancer/stomach cancer”, and “oesophageal/esophageal cancer”, were used (**Supplementary Table S1**). Articles in both English and Chinese were considered.

The identified literature was imported to EndNote, a standard software for publishing and managing bibliographies, citations, and references. Two researchers (S.H. and Y.G.) independently screened the title and abstract of each reference. Non-metabolomic (proteomic, glycomic and volatile organic compound-related) studies and conference abstracts were excluded. Studies comparing the metabolomic profiling of human biological specimens from GC/EC patients to those of control samples (either biological specimens from independent individuals or tumor-adjacent tissues) were included. Owing to our primary interest in the risk of UGI cancer development, studies concentrating on metabolomics associated with responses to cancer therapy and recurrence and metastasis of UGI cancers were additionally excluded.

For all selected articles, information on authors, publication year, sample type, analytical platform, sample size, and differentially distributed metabolites across comparison groups were independently extracted by two investigators (S.H. and Y.G.). In addition to individual metabolites, the two investigators independently reviewed findings on alterations in major metabolic pathways associated with UGI cancers.

### Study quality assessment

The quality of included studies was assessed using the Newcastle–Ottawa Scale (NOS)^17^, which covers three key domains, including Selection (4 items), Comparability (1 item), and Exposure (3 items), with a total of 8 items. Studies were rated on each of the eight items using a star system, with the final scores for each study ranging from 0 to 9 stars. A maximum of 1 star could be awarded for each item within the Selection and Exposure categories and a maximum of 2 stars allowed for the one item within the Comparability category. Studies that scored more than 6 stars were classified as high quality, and any discrepancies between the findings of the two investigators (S.H. and Y.G.) were resolved by discussion. In addition to NOS, we applied a new quality appraisal tool for cross-sectional studies using biomarker data (BIOCROSS)^18^ as a supplement. BIOCROSS includes 10 items in 5 domains, including “Study rational”, “Design/Methods”, “Data analysis”, “Data interpretation” and “Biomarker measurement”, and has been proved to be reliable in facilitating comprehensive review of human biomarker studies^18^.

## Results

### Study characteristics

**Figure 1:**
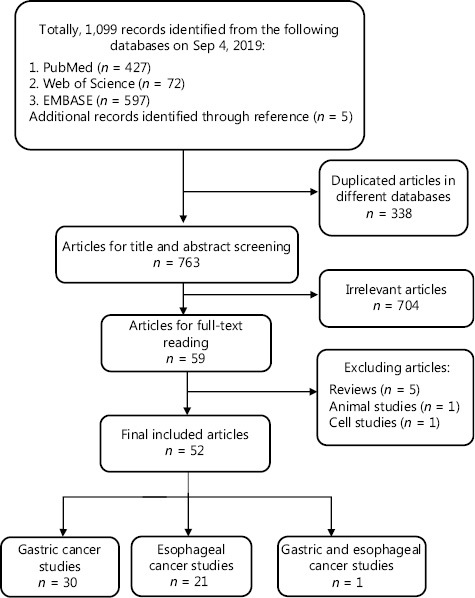
Flow chart of literature identification and the selection process.

Following application of inclusion criteria, a total of 52 studies were enrolled, including 30 on GC, 21 on EC, and 1 on both GC and EC (**Figure 1**, **Table 1**). In the majority of studies, controls were described as healthy individuals. Several studies (*n* = 10) included cases of benign gastric or esophageal lesions as controls. Among these, 5 included subjects with precancerous gastric lesions, 3 of which reported metabolic changes in precancerous gastric lesions compared with less severe lesions or normal controls, and 4 included subjects with precancerous esophageal lesions displaying metabolic alterations. However, findings from these studies were inconsistent.

The sample sizes of included studies ranged from 16 to 179, with a median of 81. Previous reports assayed tissue (*n* = 23), blood (*n* = 27), urine (*n* = 8) and gastric juice (*n* = 1), with 6 studies involving two or more types of biological specimens. The analytical platforms for measurement of metabolites also differed across studies, including nuclear magnetic resonance (*n* = 14), liquid chromatography–mass spectrometry (*n* = 20), gas chromatography–mass spectrometry (*n* = 13), capillary electrophoresis–mass spectrometry (*n* = 4), magnetic resonance spectroscopy (*n* = 2), and matrix-assisted laser desorption/ionization mass spectrometry (*n* = 1).

Review of the methods used for data analysis showed that half (*n* = 26) of the previous studies only conducted univariate tests (**Supplementary Figure S1**). Only six studies corrected for multiple comparisons, with calculation of the false discovery rate in all cases. A receiver operating characteristic (ROC) curve was plotted to delineate the performance of biomarkers, with area under the receiver operating characteristic (AUC), sensitivity, and specificity reported in 40.4% (*n* = 21) studies.

### Quality assessment of studies

**Figure 2:**
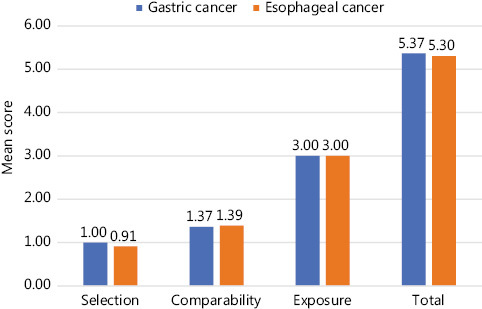
Quality assessment of included studies using the Newcastle–Ottawa Scale (NOS) (maximum scores of 4, 2, and 3 given in selection, comparability, and exposure categories, respectively).

Quality assessment with NOS revealed a mean score of 5.37 (ranging from 4 to 8) for studies on GC and 5.30 (ranging from 4 to 7) for studies on EC (**Figure 2** and **Supplementary Table S2**). The majority of studies involved hospital-based subject selection but the comparability of groups was not adequately described. Around 59.6% (31/52) of the studies considered 1 or 2 important confounding factors (mostly age or sex) during study design or statistical analysis. Quality assessment using BIOCROSS disclosed similar results to those obtained with NOS assessment, raising possible concerns on study population representativeness, study limitations, and biomarker data modeling (**Supplementary Figure S2**).

### Carbohydrate metabolism

Metabolites of carbohydrate metabolism have been previously associated with UGI cancers (**Table 2**). Several metabolites involved in cellular respiration, including lactic acid, glucose, citrate, and fumaric acid, have been frequently reported, but these results are not consistent across studies. Moreover, opposite associations of some metabolites with UGI cancers are documented by different research groups. For example, lactic acid was found to be upregulated in tissue and urine samples of GC in 8 studies^33,39,41,42,49,52,63,66^, while one group reported upregulation in tissue and conversely, downregulation in plasma^57^. Upregulation of citrate in EC was reported in 5 studies^29,54,55,60,65^ and downregulation in one study^22^. A separate study showed upregulation of citrate in plasma but downregulation in urine^53^. The findings also support distinct associations of different carbohydrate metabolites with GC and EC. Analysis of earlier data revealed upregulation of α-ketoglutaric acid in both GC^42,49,59^ and EC^29^ while isocitric acid was upregulated in GC^63^ and downregulated in EC^22^. Upregulation of glyceraldehyde in GC was reported by a number of studies^63,66^ but its association with EC is currently unclear.

We additionally attempted to provide a systematic summary of reports on metabolic pathways or profiles within the scope of carbohydrate metabolism but uncovered no direct findings on pathway-level associations or profiles.

### Amino acid metabolism

Alterations in essential and non-essential amino acids were reported for UGI cancers (**Table 3**), the most frequent being valine, glutamate, and glutamine. Increased valine was consistently detected in studies on GC based on tissue, plasma, serum, and urine samples while inconsistent findings were obtained from studies on EC. Increased glutamate in tissue, blood and urine samples was reported in the majority of available studies on GC (5/6) and EC (8/9). Earlier studies on glutamine reported variable findings from different biological specimens of GC and EC. In addition, alterations in tryptophan were frequently reported in UGI cancers. Decreased tryptophan in tissue and blood samples was observed in the majority of available studies on GC (5/6 studies) while results for EC differed based on the biosample type.

Several studies additionally reported altered levels of primary derivatives of amino acids in UGI cancers. Upregulation of kynurenine, anthranilic acid, and nicotinic acid was observed in tissue, plasma, serum and gastric juice of GC and (or) EC patients^26,32,35^. In addition, kynurenic acid was upregulated in tissue of EC and gastric juice of GC patients^29,32^ but downregulated in serum of GC patients^32^.

Although a number of studies briefly discussed the potential biological mechanisms of related amino acids^24,37,42,46,48,54,59,66^, none directly examined the pathways or profiles of amino acids associated with UGI cancers using statistical approaches.

### Lipid metabolism

All studies based on tissues, blood, and urine samples demonstrated lipid dysregulation in UGI cancers (**Table 4**), among which sphingomyelins, phosphatidylcholines, and phosphatidylethanolamine were the most frequently reported. However, findings on these lipid metabolites were not consistent across studies. In contrast, data obtained for several less commonly reported metabolites were generally consistent across studies, including upregulated triacylglycerides (2/2 studies)^68,69^ and downregulated arachidonic acid (2/2 studies)^56,59^ in GC, as well as downregulated unsaturated lipids (4/4 studies)^21,43,44,48^, low-density lipoprotein (3/3 studies) and very low-density lipoprotein (3/3 studies) in EC^21,43,44^.

Metabolites of free fatty acid (FFA) oxidation are additionally known to be associated with UGI cancers. Three studies demonstrated increased aldehyde levels in UGI cancer tissues, i.e., glyceraldehyde in GC^63,66^ and betaine aldehyde in EC^22^. In addition, 2 of the 3 endogenous ketones, acetone^22,33,39,48,53,55,65^ and β-hydroxybutyrate^33,46,52,54,60^, were reported to be associated with GC and EC, although these findings were not consistent across studies.

Despite several investigations on the mechanisms underlying altered lipid metabolism in association with UGI cancers^37,42,46,48,54,59^, we uncovered no direct findings in terms of metabolic pathway-level associations with UGI cancers.

### Nucleotide metabolism

Several studies focused on metabolites of nucleotides associated with GC and EC. Review of the data showed upregulation of pyrimidine nucleotides^67^, adenine^48,62^, and uridine-containing compounds^62^ and downregulation of uracil^48^ in EC tissues, compared with controls. In addition, one study reported upregulation of guanosine, cytidine and adenosine-containing compounds, along with downregulation of uridine in serum of EC patients^64^. In GC patients, increased levels of cytidine-containing compounds^40^ in urine and uracil in tissues and decreased uridine in tissues^52^ were documented. Although dysregulation of pathways of nucleotide metabolism in UGI cancers have been demonstrated^66^, direct evidence on the metabolic pathways of nucleotides related to UGI cancers is still unavailable.

### Performance of metabolites as potential biomarkers

A total of 21 studies assessed the performance of specific metabolites as potential biomarkers for predicting the risk of UGI cancers, the majority of which showed area under ROC curve (AUC) values ≥ 0.80 (19/21 studies) for individual metabolites or metabolite set. However, among the predictive models reported, we identified no overlapping metabolite biomarkers for risk of UGI cancers.

## Discussion

Metabolomic profiling has been increasingly applied for comprehensive characterization of the functional phenotypes of metabolic changes in cancers. Here, we systematically reviewed 52 molecular epidemiologic studies on metabolomic profiling of human UGI cancers and summarized key findings on the dysregulation of major metabolic pathways (glucose, amino acid, lipid, and nucleotide) associated with UGI cancers.

Metabolic reprogramming is a hallmark of cancer^71^. During tumor development and progression, metabolic pathways are reprogrammed to maintain cancer cell proliferation and survival, which involves large demands for adenosine triphosphate (ATP), nicotinamide adenine dinucleotide phosphate, nicotinamide adenine dinucleotide, carbon skeletons, and other molecules^72^. Metabolic alterations have been shown to be closely associated with GC and EC, raising the profile of metabolomics as a promising tool for etiologic research and biomarker screening of UGI cancers^4,12^.

Alterations in carbohydrate metabolism have been reported in a number of earlier metabolomic studies on UGI cancers. Detection of dysregulated pivotal intermediates of glycolysis, such as glucose, fructose, glyceraldehyde, and pyruvic acid, in the UGI cancers GC and EC^29,53,63,66^ corroborates the well-known Warburg effect^22,29,33^, which highlights the phenomenon that most cancer cells avidly consume glucose to generate energy mainly by glycolysis instead of oxidative phosphorylation through the tricarboxylic acid (TCA) cycle, even under aerobic conditions^73^. This less efficient method of generating ATP by glycolysis is subsequently rationalized through diverting available glycolytic intermediates into biosynthetic pathways critical for the synthesis of amino acids, lipids and nucleosides to produce new cells^74^. The Warburg effect can also enhance generation of pyruvic acid and subsequent lactic acid fermentation catalyzed by lactate dehydrogenase, which may partly explain the upregulation of lactic acid observed particularly in GC.

The metabolite intermediates of the TCA cycle, such as citrate, α-ketoglutaric acid, and fumaric acid, have also been identified in UGI cancers^22,29,30,33,37,39,41,42,49,51–55,57,59,60,63,65,66^. Consistent with the findings of a previous systematic review^4^, fumaric acid and citrate were determined as the most frequently reported metabolites of the TCA cycle. However, results from different studies were inconsistent, supporting the necessity for further investigation. Although cancer cells favor glycolysis over oxidative phosphorylation, the increase in TCA cycle metabolites may rely on the process of anaplerosis, which refers to replenishment of TCA metabolites via generation of α-ketoglutarate from the source of glutamate by deaminating glutamine^72,75^. The present review revealed frequent aberrant metabolism of glutamine and glutamate in UGI cancer patients, supporting the importance of the pathway in this cancer type.

Along with glutamine and glutamate, valine is another frequently reported amino acid dysregulated in UGI cancer ^20,22,26,29,33,39,48^. Elevated levels of valine can be converted into TCA intermediates to generate energy^12^. The collective data from studies in the literature clearly demonstrate that valine is upregulated in GC^20,33,39,41,51,61,66^ although findings in EC are inconsistent^22,26,29,48,53,55,65,67^. It remains to be established whether these results reflect differential metabolic profiling for GC and EC.

Prior studies have also reported alterations in tryptophan and kynurenine in UGI cancers^26,32,35,46,54^, indicating that potential metabolic perturbations of the tryptophan/kynurenine catabolism pathway are associated with development of EC and GC. Considerable evidence supports the theory that molecules in this pathway are involved in the immune regulation of tumor cells. For example, the tryptophan-catabolizing enzyme, indoleamine-2, 3-dioxygenase (IDO), may alter tumor microenvironment to favor cancer progression^76,77^. IDO is proposed to function as an immune suppressor and induce immune tolerance^76,78^, and its increased expression in the tumor microenvironment is correlated with immunosuppression in UGI cancers^32,79^. A number of studies have reported upregulation of lysine, serine or arginine^22,24,39,66,67^ and downregulation of isoleucine, tyrosine or glycine^20,23,52,55,65^ in UGI cancers, although the findings were not all consistent^20,29,54^. Increased levels of amino acids in tissues and other biological specimens may be generated from various sources, such as environmental degradation of the extracellular matrix and autophagic degradation of preexisting intracellular proteins^66^, while amino acid overutilization in tumor tissues may have contributed to the decreased levels of amino acids (such as methionine, histidine, and leucine) observed in some studies^33,57^.

While previous investigations have highlighted changes in fatty acid metabolic pathways associated with UGI cancers, mixed findings were reported on the levels of unsaturated and saturated FFA in UGI cancers^48,59^. Low levels of FFA or lipids have been attributed to increased consumption by tumors due to their anabolic metabolism while metabolic reprogramming in cancer is related to fatty acid increase in the tumor environment^80^. In addition, systemic lipolysis secondary to cancer cachexia or *de novo* fatty acid synthesis may contribute to FFA accumulation^4,81^. Although the mechanisms underlying lipid synthesis in cancer are not fully understood at present, it is proposed that *de novo* lipid synthesis leads to the formation of structural lipids for cell membrane production, provides energy through β-oxidation, and affects fundamental cellular processes, such as signal transduction^81,82^.

β-Oxidation of fatty acids is a main source of energy generation^83^. Dysregulation of aldehydes and ketones, the metabolic products of β-oxidation, has been consistently reported in UGI cancer patients^22,33,39,46,48,52–55,60,63,65,66^. Altered ketone body synthesis and degradation have also been documented in relation to UGI cancers^42,46,54^. Fatty acid β-oxidation not only efficiently produces energy but also promotes reactive oxygen species generation^84^, facilitating lipid peroxidation and aldehyde production^85^.

Several studies have shown alterations in nucleotide metabolites in UGI cancers^40,48,52,62,64,66,67^. Nucleotide synthesis and metabolism are required for adequate energy generation and proposed to be critical for proliferation and differentiation of cancer cells^12^. The growth superiority of cancer cells gradually switching to anaerobic glycolysis may partly explain the mixed findings on nucleotide metabolites.

Review of the collective data from the literature suggests that consistent findings on UGI cancer-associated metabolites based on metabolomic studies are limited. Moreover, discrepancies in results even exist among studies on the same types of biospecimens. While different analytical platforms and biospecimens may partly explain these inconsistencies, discrepant findings also reflect the heterogeneity in study design and subject selection across studies. The majority of prior studies were hospital-based cross-sectional or case-control analyses with a modest sample size and may have led to high false-positive probability due to negligence of potential multiple comparisons and lack of an independent replication group. Under representativeness of specific study populations may have restricted the extrapolation of findings across studies. Multivariate adjustments were not possible for most studies due to the unavailability of detailed information on UGI cancer risk factors and potential residual confounding may have distorted these findings.

## Conclusions

In conclusion, a total of 52 molecular epidemiologic studies on metabolomics have been conducted for human UGI cancers over the years. Studies on metabolomics have thus far facilitated effective biomarker detection in GC and EC, supporting the potential of applying metabolomic profiling in cancer prevention and management efforts. Although a number of metabolites have been identified for GC and EC, identification of putative metabolomic biomarkers has been inadequate. Application of metabolomic profiling to molecular epidemiologic studies on UGI cancers may provide insights into the biological significance of crucial metabolites and metabolic pathways but there is no actual information on the underlying mechanisms. Given the multi-stage progression of UGI carcinogenesis, it is necessary to identify metabolic biomarkers associated with both precancerous and early UGI cancers, which would benefit screening of high-risk populations and early diagnosis. Limited studies to date have focused on metabolomic profiling for the cascade of precancerous lesions and UGI cancers.

To fulfill the potential of effectively applying metabolomics for UGI cancer prevention and control in public health and clinical practices, major gaps need to be filled with the aid of well-designed molecular epidemiologic studies. Studies with large sample sizes, clearly defined study population and independent validation samples are warranted to identify metabolomic biomarkers and define the critical metabolomic pathways and patterns. Prospective follow-up of subjects covering a cascade of precancerous lesions and subsequent cancers would also be advantageous in identifying metabolomics biomarkers for efficient assessment of the risk of UGI cancer development and progression.

## Figures and Tables

**Table 1:** Characteristics of the included studies

Study	Region	Sample type	Analytical platform	Cancer group		Control group	
				Cancer	Sample size	Control	Sample size
Lee (Anal Chim Acta.) 2019^19^	South Korea	Plasma	nUHPLC–MS/MS	GC	20	Healthy subjects	20
Xiu (Acad J Second Military Medical Univ.) 2018^20^	Mainland China	Plasma	UHPLC–MS/MS	GC	104	Healthy subjects	50
Corona (Int J Mol Sci.) 2018^21^	Italy	Serum	LC–MS/MS	GC Training set (*n* = 49) Validation set (*n* = 22)	71	First-degree relatives Training set (*n* = 37) Validation set (*n* = 17)	54
Tokunaga (Int J Oncol.) 2018^22^	Japan	Tissue	CE–TOFMS	EC	35	Tumor-adjacent tissues	35
Jing (Iubmb Life.) 2018^23^	Mainland China	Plasma	LC–MS/MS	EC	84	Gastric ulcer	82
Ma (J Pharm Biomed Anal.) 2018^24^	Mainland China	Serum	2D LC–MS	EC	34	Healthy subjects	32
Lario (Sci Rep.) 2017^25^	Spain	Plasma	LC–MS	GC	20	NAG- (*n* = 19); CAG+ (*n* = 20); PLGC- (*n* = 19)	60
Zhang (Biochem Biophys Res Commun.) 2017^26^	Mainland China	Tissue	LC/MS	EC Training set (*n* = 35) Validation set (*n* = 5)	40	Tumor-adjacent tissues Training set (*n* = 35) Validation set (*n* = 5)	40
Cheng (Biochem Biophys Res Commun.) 2017^27^	Mainland China	Serum	UPLC–MS/MS	EC (*n* = 38) Metastatic EC (*n* = 38)	76	Healthy subjects	28
Cheng (Comb Chem High Throughput Screen.) 2017^28^	Mainland China	Serum	LC–MS/MS	EC Test set (*n* = 5) Training set (*n* = 35)	40	Healthy subjects Test set (*n* = 5) Training set (*n* = 22)	27
Zhu (Gastroenterol Res Pract.) 2017^29^	Mainland China	Tissue	GC/TOF–MS	EC Serum (*n* = 24) Tissue (*n* = 19)	43	Healthy subjects (serum, *n* = 21) and tumor-adjacent tissues (tissue, *n* = 19)	40
Reed (Neoplasia.) 2017^30^	UK	Tissue	^1^H NMR	EC	46	BO, *n* = 7); patients undergoing upper gastrointestinal endoscopy for dyspeptic symptoms but without endoscopic abnormalities (controls, *n* = 68)	75
Wang (Oncotarget.) 2017^31^	Mainland China	Serum	HPLCESI/Q-TOFMS	GC Test group (*n* = 24) Validation group (*n* = 14) Additional group (*n* = 87)	125	Healthy subjects Test group (*n* = 24) Validation group (*n* = 14)	38
Choi (Biomed Chromatogr.) 2016^32^	South Korea	Serum and gastric juice	LC–MS/MS	GC	35	Gastritis (same race and same geo-graphic area)	17
Wang (BMC Cancer.) 2016^33^	Mainland China	Tissue	^1^H NMR	GC	125	Healthy subjects	54
Chan (Br J Cancer.) 2016^34^	Canada	Urine	^1^H-NMR	GC	43	Benign gastric disease (*n* = 40) and healthy subjects (*n* = 40)	80
Kuligowski (J Proteome Res) 2016^35^	Spain	Plasma	UPLC–TOFMS	GC	33	Dyspepsia	110
Xu (Sci Rep.) 2016^36^	Mainland China	Urine	LC–MS	EC	62	Healthy subjects	62
Liang (Appl Biochem Biotechnol.) 2015^37^	Mainland China	Urine	LC–MS	GC	13	Healthy subjects	9
Mir (J Proteomics.) 2015^38^	India	Serum	LC–MS	EC	40	Healthy subjects	10
Jung (Ann Surg Oncol.) 2014^39^	South Korea	Urine and tissue	^1^H NMR and HR-MAS NMR	GC Urine (*n* = 50) Tissue (*n* = 30)	80	Healthy subjects Urine (*n* = 50) Tissue (*n* = 30)	80
Lo (Clin Chim Acta.) 2014^40^	Taiwan	Urine	HPLC/ESI–MS/MS	GC	49	Healthy subjects	40
Chen (Electrophoresis.) 2014^41^	Mainland China	Urine	MRB–CE–MS	GC	26	Healthy subjects	14
Hur (PLoS One.) 2014^42^	South Korea	Tissue	GC–MS	GC	45	Tumor-adjacent tissue	45
Yang (Se Pu.) 2014^43^	Mainland China	Serum	LC–MS	GC	20	Healthy subjects	40
Kwon (Open Proteomics J.) 2014^44^	South Korea	Tissue	MALDI MS	GC	12	Tumor-adjacent tissue	12
Yang (Anal Bioanal Chem.) 2013^45^	Mainland China	Tissue	^1^H NMR	EC	17	Tumor-adjacent tissue	14
Zhang X (Biochim Biophys Acta) 2013^46^	Mainland China	Serum	^1^H NMR and UHPLC	EC	25	Healthy subjects	25
Liu (Int J Mol Sci.) 2013^47^	Mainland China	Plasma	UPLC/TOF/MS	EC	53	Healthy subjects	53
Wang (Molecular Cancer.) 2013^48^	Mainland China	Tissue	^1^H NMR	EC	89	Tumor-adjacent tissue	26
Song (Chinese J Clin Nutrition.) 2013^49^	Mainland China	Serum and tissue	GC–MS	GC Tissue (*n* = 40) Serum (*n* = 40)	80	Tumor-adjacent tissue (*n* = 40) and serum from healthy subjects (*n* = 40)	80
Ikeda (Biomed Chromatogr.) 2012^50^	Japan	Serum	GC–MS	GC (*n* = 11) and EC (*n* = 15)	26	Healthy subjects	12
Song (Braz J Med Biol Res.) 2012^51^	Mainland China	Serum	GC–MS	GC	30	Healthy subjects	30
Aa (Metabolomics.) 2012^52^	Mainland China	Serum and tissue	GC/TOFMS	GC (*n* = 17) and postoperative GC (*n* = 15)	32	Chronic superficial gastritis	20
Hasim (Mol Biol Rep.) 2012^53^	Mainland China	Plasma and urine	NMR	EC	108	Healthy subjects	40
Zhang (PLoS One.) 2012^54^	US	Serum	LC-MS and NMR	EC	67	Healthy subjects (*n* = 34), BE, *n* = 3), and high-grade dysplasia (HGD, *n* = 9)	46
Davis (World J Surg Oncol.) 2012^55^	Canada	Urine	^1^H NMR	EC	44	Healthy subjects (*n* = 75) and BE (*n* = 31),	106
Song (Chin Med J.) 2012^56^	Mainland China	Tissue	GC/MS	GC	30	Tumor-adjacent tissues	30
Sun (Chinese J Gastroenterol.) 2011^57^	Mainland China	Tissue and plasma	GC/TOF–MS	GC tissue (*n* = 17), GC plasma (*n* = 15), and postoperative GC plasma (*n* = 15)	47	Chronic superficial gastritis tissue (*n* = 20) and plasma (*n* = 15)	35
Yu (J Gastroenterol Hepatol.) 2011^58^	Mainland China	Plasma	GC/TOF–MS	GC	22	Chronic superficial gastritis (*n* = 19), chronic atrophic gastritis (*n* = 13), intestinal metaplasia (*n* = 10), and dysplasia (*n* = 15)	57
Song (Oncol Rep.) 2011^59^	Mainland China	Tissue	GC–MS	GC	30	Tumor-adjacent tissue	30
Zhang (J Thorac Cardiovasc Surg.) 2011^60^	US	Serum	NMR	EC	68	Healthy subjects (*n* = 34), BE (*n* = 5), and HGD (*n* = 11)	50
Wu (Anal Bioanal Chem.) 2010^61^	Mainland China	Tissue	GC–MS	GC	18	Tumor-adjacent tissue	18
Yakoub (Cancer Res.) 2010^62^	UK	Tissue	NMR	EC	52	Healthy subjects	35
Cai (Mol Cell Proteomics.) 2010^63^	Mainland China	Tissue	GC–MS	GC	65	Tumor-adjacent tissue	65
Djukovic (Rapid Commun Mass Spectrom.) 2010^64^	US	Serum	HPLC/TQMS	EC	14	Healthy subjects	12
Ayshamgul (Chin J Oncol.) 2010^65^	Mainland China	Plasma	^1^H NMR	EC	109	Healthy subjects	50
Hirayama (Cancer Res.) 2009^66^	Japan	Tissue	CE–MS	GC	12	Tumor-adjacent tissue	12
Wu (J Chromatogr B.) 2009^67^	Mainland China	Tissue	GC/MS	EC	20	Tumor-adjacent tissues	20
Calabrese (Cancer Epidemiol Biomarkers Prev.) 2008^68^	Italy	Tissue	HR-MAS MRS	GC	5	Healthy subjects (*n* = 12), autoimmune atrophic gastritis (*n* = 5), and *H. pylori* infection (*n* = 5)	22
Tugnoli (Oncol Rep.) 2006^69^	Italy	Tissue	HR-MAS NMR	GC	5	Healthy subjects	11
Mun (Magn Reson Imaging.) 2004^70^	South Korea	Tissue	^1^H MRS	GC	13	Tumor-adjacent tissue	22

BO, Barrett’s esophagus; CAG, chronic active gastritis; GC, gastric cancer; EC, esophageal cancer; HGD, high grade dysplasia; NAG, non-active gastritis; PLGC, precursor lesions of gastric cancer; *H. pylori*, *Helicobacter pylori*; ^1^H NMR, proton nuclear magnetic resonance; HR-MAS–NMR, high resolution-magic angle spinning-nuclear magnetic resonance spectroscopy; GC–MS, gas chromatography–mass spectrometry; CE–MS, capillary electrophoresis–mass spectrometry; HPLC–MS, high-performance liquid chromatography–mass spectrometry; LC–MS, liquid chromatography–mass spectrometry

**Table 2:** Changes in carbohydrate metabolites in GC and EC, compared with controls

Study	Sample type	Analytical platform	Glycolysis				Anaerobic respiration lactic acid/lactate	TCA cycle					
			Glucose	Fructose	Glyceraldehyde	Pyruvic acid		Citrate	Isocitric acid	α-ketoglutaric acid/α-ketoglutarate	Succinate	Fumaric acid/fumarate	Malate
GC													
Hirayama (2009)^66^	Tissue	CE–MS	↑	↑	↑		↑	↓				↑	↑
Cai (2010)^63^	Tissue	CE–MS		↑	↑	↑	↑		↑			↓	
Sun (2011)^57^	Tissue Plasma	GC/TOF-MS	↓ ↓	↓			↑ ↓	↑			↓	↓	↓
Song (2011)^59^	Tissue	GC–MS								↑		↑	
Song (2012)^51^	Serum	GC–MS										↓	
Aa (2012)^52^	Serum Tissue	GC/TOFMS	↓ ↓				↑	↑			↓	↓	↓ ↑
Ikeda (2012)^50^	Serum	GC–MS				↓							
Song (2013)^49^	Serum Tissue	GC–MS					↑			↑		↓ ↑	
Jung (2014)^39^	Urine Tissue	^1^H NMR; HR-MAS NMR					↑ ↑				↑		
Chen (2014)^41^	Urine	MRB-CE-MS					↑	↓			↓		↓
Hur (2014)^42^	Tissue	GC-MS				↑	↑			↑	↑	↑	↑
Liang (2015)^37^	Urine	LC-MS						↑			↓		↑
Wang (2016)^33^	Tissue	^1^H NMR	↓				↑				↑	↑	
Wang (2017)^31^	Serum	HPLCESI/Q-TOFMS	↑										
EC													
Ayshamgul (2010)^65^	Plasma	^1^H NMR	↑				↓	↑					
Zhang (2011)^60^	Serum	NMR	↑				↑	↑					
Zhang (2012)^54^	Serum	LC-MS and NMR	↑				↑	↑					
Hasim (2012)^53^	Urine Plasma	^1^H NMR	↑ ↑			↑	↓	↓ ↑					
Davis (2012)^55^	Urine	^1^H NMR	↓				↓	↑			↓		
Ikeda (2012)^50^	Serum	GC–MS				↓							
Zhang (2013)^46^	Serum	^1^H NMR; UHPLC	↓				↑						
Liu (2013)^47^	Plasma												
Wang (2013)^48^	Tissue	^1^H NMR	↓										
Zhu (2017)^29^	Serum Tissue	GC/TOF-MS	↓ ↓			↑ ↓	↑ ↓	↑ ↑		↑ ↑	↓ ↑	↑ ↑	↑ ↑
Reed (2017)^30^	Tissue	^1^H NMR										↓	
Tokunaga M (2018)^22^	Tissue	CE-TOFMS				↓	↑	↓	↓			↓	

EC, esophageal cancer; GC, gastric cancer; TCA, tricarboxylic acid; ^1^H NMR, proton nuclear magnetic resonance; HR-MAS–NMR, High resolution-magic angle spinning-nuclear magnetic resonance spectroscopy; GC–MS, gas chromatography–mass spectrometry; CE–MS, capillary electrophoresis–mass spectrometry; HPLC–MS, high-performance liquid chromatography–mass spectrometry; LC–MS, liquid chromatography–mass spectrometry

**Table 3:** Changes in amino acid metabolites in GC and EC, compared with controls

Study	Sample type	Analytical platform	Essential amino acid									Non-essential amino acid										
			Val	His	Phe	Thr	Leu	Met	Trp	Ile	Lys	Tyr	Gln	Glu	Ser	Asn	Gly	Ala	Arg	Asp	Cys	Pro
GC																						
Tugnoli (2006)^69^	Tissue	HR-MAS NMR															↑					
Calabrese (2008)^68^	Tissue	HR-MAS MRS															↑	↑				
Hirayama (2009)^66^	Tissue	CE–MS	↑	↑	↑	↑	↑	↑	↑	↑	↑	↑		↑	↑	↑	↑	↑	↑		↑	↑
Wu (2010)^61^	Tissue	GC–MS	↑							↑			↑		↑							
Yu (2011)^58^	Plasma	GC/TOF MS												↑		↑						
Song (2012)^51^	Serum	GC–MS	↑										↓									
Aa (2012)^52^	Serum Tissue	GC/TOFMS													↓		↓			↓ ↑		
Jung (2014)^39^	Urine Tissue	1H NMR; HR-MAS NMR	↑↑	↑	↑ ↑		↑ ↑	↑		↑	↑	↑ ↑	↑			↑	↑ ↑	↑	↑			
Chen (2014)^41^	Urine	MRB-CE-MS	↑	↓			↑	↓		↑					↓				↑	↓		
Yang (2014)^43^	Serum	LC–MS																	↑	↑		
Liang (2015)^37^	Urine	LC–MS															↓	↑				↑
Wang (2016)^33^	Tissue	^1^H NMR	↑				↑		↓	↑	↑	↑	↑	↑	↑		↓			↑		
Chan (2016)^34^	Urine	^1^H-NMR																↑				
Kuligowski (2016)^35^	Plasma	UPLC–TOFMS							↓				↑									
Wang (2017)^31^	Serum	HPLCESI/Q-TOFMS						↑					↑	↑								
Lario (2017)^25^	Plasma	LC–MS		↓					↓				↑									
Xiu (2018)^20^	Plasma	UHPLC–MS/MS	↑	↓	↓	↓	↓	↓	↓	↓	↓	↓	↓	↓	↑		↓	↓	↑	↓	↓	
Jing (2018)^23^	Plasma	LC–MS/MS		↓					↓				↓						↓			
EC																						
Wu (2009)^67^	Tissue	GC/MS	↑							↑		↑			↑			↑				
Yakoub (2010)^62^	Tissue	NMR												↑								
Ayshamgul (2010)^65^	Plasma	^1^H NMR	↓				↓			↓								↓				
Zhang (2011)^60^	Serum	NMR									↑		↑									
Zhang (2012)^54^	Serum	LC–MS and NMR					↓	↓	↓	↓	↑	↓	↑									
Hasim (2012)^53^	Urine Plasma	^1^H NMR	↓ ↓	↓	↑		↓			↓		↑		↑				↓				
Davis (2012)^55^	Urine	^1^H NMR	↓				↓		↓			↓	↓				↓	↑				
Ikeda (2012)^50^	Serum	GC–MS													↑							
Yang (2013)^45^	Tissue	^1^H NMR												↑				↑				
Zhang (2013)^46^	Serum	^1^H NMR; UHPLC		↑	↑		↑	↓	↓		↑	↓	↑	↑						↑	↑	
Wang (2013)^48^	Tissue	^1^H NMR	↑		↑		↑	↑		↑		↓	↓	↑			↓			↓		
Xu (2016)^36^	Urine	LC–MS											↑	↑								
Zhang (2017)^26^	Tissue	LC–MS	↑		↑								↓	↑	↓							↑
Cheng (2017)^27^	Serum	UPLC-MS/MS							↑													
Cheng (2017)^28^	Serum	LC-MS/MS			↓																	
Zhu (2017)^29^	Serum Tissue	GC/TOF-MS	↓ ↑	↑	↓ ↑	↓ ↑	↓ ↑	↓ ↑	↓ ↑	↓ ↑	↑ ↑	↑	↑ ↓	↓ ↑	↓ ↑		↓ ↑	↓ ↑	↓ ↑	↑	↑ ↑	↓ ↑
Reed (2017)^30^	Tissue	^1^H NMR											↓					↓				
Tokunaga (2018)^22^	Tissue	CE-TOFMS	↑		↑	↑	↑	↑	↑	↑	↑	↑	↓	↑	↑	↑	↑	↑	↑	↑	↑	↑
Ma (2018)^24^	Serum	2D LC–MS		↓			↑				↑		↑		↑				↑			

EO, esophageal cancer; GC, gastric cancer; Val, valine; His, histidine; Phe, phenylalanine; Thr, threonine; Leu, leucine; Met, methionine; Trp, tryptophan; Ile, isoleucine; Lys, lysine; Tyr, tyrosine; Gln, glutamine; Glu, glutamic acid; Ser, serine; Asn, asparagine; Gly, glycine; Ala, alanine; Arg, arginine; Asp, aspartic acid; Cys, cystine; Pro, proline; ^1^H NMR, proton nuclear magnetic resonance; HR-MAS-NMR, high-resolution magic angle spinning-nuclear magnetic resonance spectroscopy; GC–MS, gas chromatography–mass spectrometry; CE–MS, capillary electrophoresis–mass spectrometry; HPLC–MS, high-performance liquid chromatography–mass spectrometry; LC–MS, liquid chromatography–mass spectrometry

**Table 4:** Changes in lipid metabolites in gastric cancer and esophageal cancer, compared with controls

Study	Sample type	Analytical platform	Un-saturated fatty acid								Saturated fatty acid			
			Palmitoleic acid	Cervonic acid	Linoleic acid	Oleic acid	Linolenic acid	Vaccenic acid	Gondoic acid	Arachidonic acid	Myristic acid	Enanthic acid	Margaric acid	Caprylic acid
GC														
Song (2011)^59^	Tissue	GC–MS	↓			↑		↓		↓				
Yu (2011)^58^	Plasma	GC/TOF MS							↑					
Song (2012)^51^	Serum	GC–MS			↓	↓								
Aa (2012)^52^	Serum Tissue	GC/TOFMS	↑	↑								↑ ↑		
Song (2012)^56^	Tissue	GC–MS				↓				↓				
Mun (2004)^70^	Tissue	^1^H NMR	Decrease in lipids											
Tugnoli (2006)^69^	Tissue	HR-MAS NMR	Increase in TAG											
Calabrese (2008)^68^	Tissue	HR-MAS MRS	Increase in TAG											
Hirayama (2009)^66^	Tissue	CE–MS	Increase in glyceraldehyde-3P											
Cai (2010)^63^	Tissue	CE–MS	Increase in glyceraldehyde											
Jung (2014)^39^	Urine Tissue	^1^H NMR; HR-MAS NMR	Urine: increase in acetone Tissue: decrease in lipid I and lipid II											
Yang (2014)^43^	Serum	LC-MS	Increase in PE, FFA, PC, SM, dihydrocholesterol Decrease in lyso-PC, lyso-PE, FFA, PC, choline, SM											
Kwon (2014)^44^	Tissue	MALDI MS	Increase in SM Decrease in PC											
Wang (2016)^33^	Tissue	^1^H NMR	Decrease in lipids, VLDL, acetone, β-hydroxybutyrate											
Corona (2018)^21^	Serum	LC-MS/MS	Increase in acylcarnitines derivatives (C2, C16, C18:1) Decrease in hydroxylated SM (SM(OH)22:1, SM(OH)22:2, SM(OH)24:1) and PC (PC ae 40:1, PC ae 42:2, PC ae 42:3)											
Lee (2019)^19 ^	Plasma	nUHPLC-MS/MS	Increase in LPC18:2; LPE16:0 Decrease in PI; PE; LPE18:1; LPE18:0; LPC16:0; LPA18:2; PC											
EC														
Wu (2009)^67^	Tissue	GC/MS	↑								↑			
Zhang (2012)^54^	Serum	LC-MS and NMR			↓		↓				↓		↑	
Zhang (2017)^26^	Tissue	LC-MS				↑				↑				
Zhu (2017)^29^	Serum Tissue	GC/TOF-MS			↓ ↑	↑								
Ayshamgul (2010)^65^	Plasma	^1^H NMR	Decrease in unsaturated lipid, VLDL and LDL, acetone											
Zhang (2011)^60^	Serum	NMR	Increase in β-hydroxybutyrate											
Davis (2012)^55^	Urine	^1^H NMR	Decrease in acetone											
Hasim (2012)^53^	Urine Plasma	^1^H NMR	Decrease in unsaturated lipids, VLDL and LDL, acetone											
Zhang (2013)^46^	Serum	^1^H NMR; UHPLC	Increase in β-hydroxybutyrate Decrease in unsaturated lipids, VLDL and LDL											
Liu (2013)^47^	Plasma	UPLC/TOF/MS	Increase in PI, PA, PC, PE, sphinganine-1-phosphate, PS (16:0/14:0)											
Wang (2013)^48^	Tissue	^1^H NMR	Increase in short-chain fatty acids, acetone Decrease in unsaturated lipids											
Mir (2015)^38^	Serum	LC–MS	Increase in PC (20:4/0:0), PC (16:0/18:2), PC(18:1/18:1) Decrease in PC (18:2/0:0), PC (18:1/18:2), PC(O-18:1/18:2), PC(16:0/h18:2)											
Ma (2018)^24^	Serum	2D LC–MS	Decrease in fatty acids, PC, and FFA											
Tokunaga (2018)^22^	Tissue	CE-TOFMS	Increase in betaine aldehyde Decrease in dihydroxyacetone											

EC, esophageal cancer; GC, gastric cancer; TAG, triacylglycerides; PE, phosphatidylethanolamine; FFA, free fatty acids; PC, phosphatidylcholine; SM, sphingomyelin; VLDL, very low-density lipoprotein; LDL, low-density lipoprotein; PI, phosphatidylinositol; PA, phosphatidic acid; PS, phosphatidylserine; ^1^H NMR, proton nuclear magnetic resonance; HR-MAS-NMR, high resolution-magic angle spinning-nuclear magnetic resonance spectroscopy; GC–MS, gas chromatography–mass spectrometry; CE–MS, capillary electrophoresis–mass spectrometry; HPLC–MS, high-performance liquid chromatography–mass spectrometry; LC–MS, liquid chromatography–mass spectrometry
