# Supplementary material for: A systematic review of metabolomic profiling of gastric cancer and esophageal cancer
Source: Cancer Biol Med. 2020 Feb 15;17(1):181–98. doi: 10.20892/j.issn.2095-3941.2019.0348 (PMC7142846; doi:10.20892/j.issn.2095-3941.2019.0348)
Supplement: Supplementary file 1 [file cbm-17-181-s001.pdf]

Supplementary materials

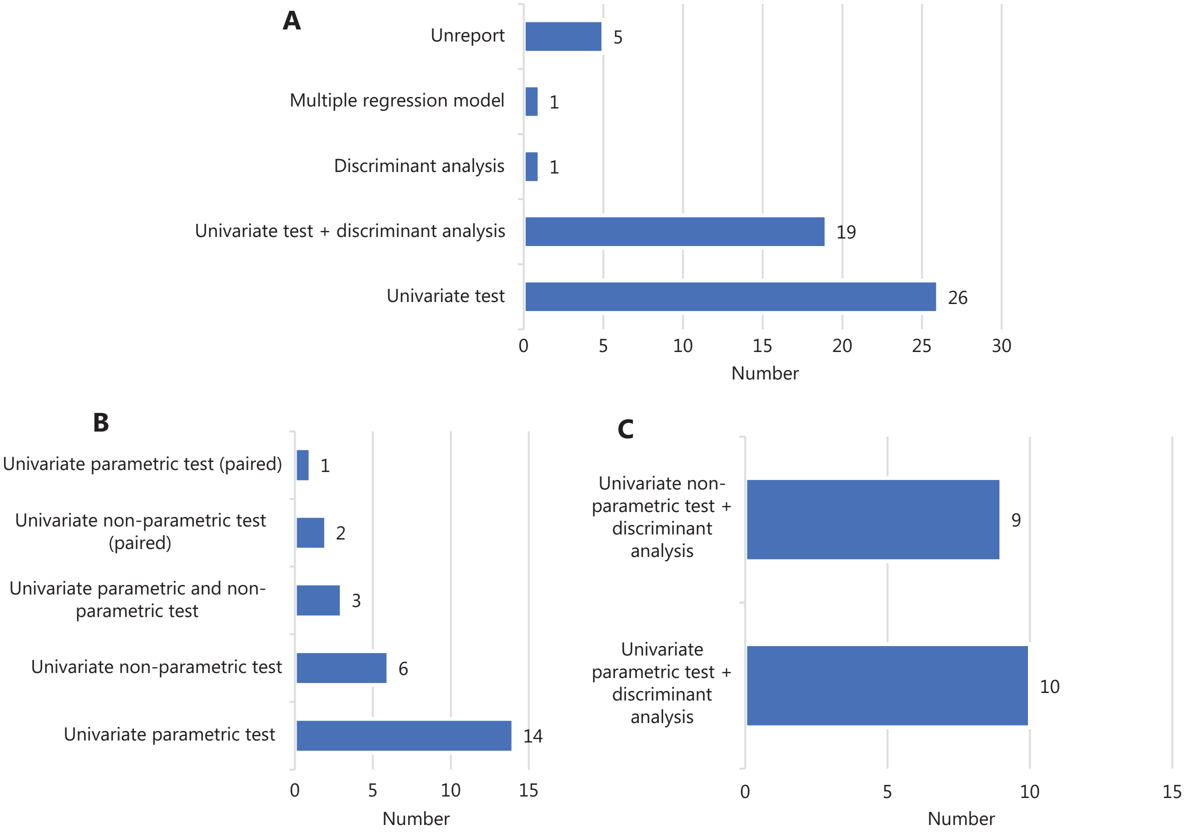

Figure S1 Summary of the biomarker detection methods used in the included studies. (A) Biomarker detection methods used. (B) Univariate test methods. (C) Combination of univariate and discriminant analyses.

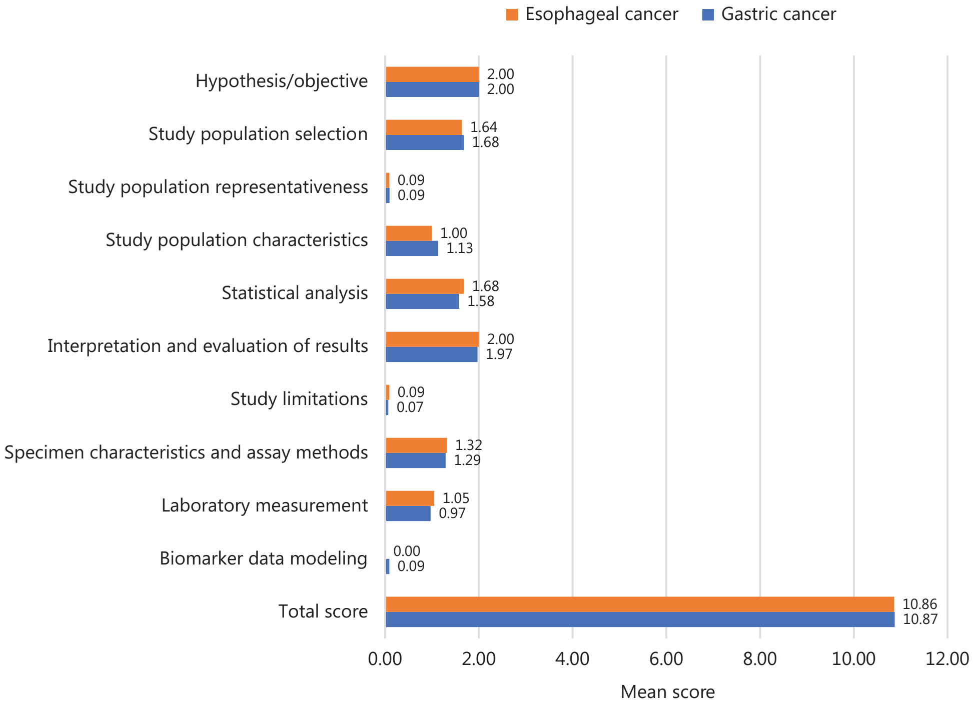

Figure S2 Quality assessment of included studies using BIOCROSS.

Table S1 Summary of literature search strategies

Database	Search strategies	Results
PubMed	[mass spectrometry (Title/Abstract) OR nuclear magnetic resonance spectroscopy (Title/Abstract) OR metabolomics (Title/Abstract) OR metabonomic (Title/Abstract) OR metabolic profiling (Title/Abstract)] AND [stomach neoplasms (MeSH Terms) OR esophageal neoplasms (MeSH Terms)]	427
Web of Science	[TI = (mass spectrometry) OR TI = (nuclear magnetic resonance spectroscopy) OR TI = metabolomic OR TI = metabonomic OR TI = (metabolic profiling)] AND [TI = (stomach neoplasms) OR TI = (gastric cancer) OR TI = (stomach cancer) OR TI = (gastric carcinoma) OR TI = (esophageal neoplasms) OR TI = (esophageal cancer) OR TI = (carcinoma of esophagus) OR TI = (esophageal carcinoma) OR TI = (esophagus cancer)]	72
EMBASE	(“mass spectrometry”:ab OR “nuclear magnetic resonance spectroscopy”:ab OR metabolomic:ab OR metabonomic:ab OR “metabolic profiling”:ab) AND (“stomach neoplasms”:ab OR “gastric cancer”:ab OR “stomach cancer”:ab OR “gastric carcinoma”:ab OR “esophageal neoplasms”:ab OR “esophageal cancer”:ab OR “carcinoma of esophagus”:ab OR “esophageal carcinoma”:ab OR “esophagus cancer”:ab)	597

Table S2 Quality assessment of included studies using the Newcastle–Ottawa Scale (NOS)

Study	Group	Selection				Comparability	Exposure			Total
		Adequate definition of cases	Representativeness of the cases	Selection of controls	Definition of controls	Comparability of cases and controls*	Ascertainment of exposure	Same method of ascertainment for cases and controls	Non-response rate	
Lee GB (Anal Chim Acta) 2019	GC	☆	★	★	★	★★	★	★	★	8
Xiu FM (Academic Journal of Second Military Medical University) 2018	GC	☆	☆	☆	★	★☆	★	★	★	5
Corona G (Int J Mol Sci) 2018	GC	★	★	★	★	★☆	★	★	★	8
Tokunaga M (Int J Oncol) 2018	EC	☆	☆	☆	☆	★★	★	★	★	5
Jing FY (Iubmb Life) 2018	EC	☆	☆	☆	☆	★★	★	★	★	5
Ma W (J Pharm Biomed Anal) 2018	EC	☆	☆	☆	★	★★	★	★	★	6
Lario, S (Sci Rep) 2017	GC	★	★	★	★	☆★	★	★	★	8
Zhang H (Biochem Biophys Res Commun) 2017	EC	★	☆	☆	☆	★★	★	★	★	6
Cheng J (Biochem Biophys Res Commun) 2017	EC	☆	☆	☆	★	★★	★	★	★	6
Cheng J (Comb Chem High Throughput Screen) 2017	EC	★	☆	☆	★	★★	★	★	★	7
Zhu X (Gastroenterol Res Pract) 2017	EC	☆	☆	☆	☆	★☆	★	★	★	4
Reed MA (Neoplasia) 2017	EC	☆	☆	☆	★	☆☆	★	★	★	4
Wang DG (Oncotarget) 2017	GC	★	☆	☆	★	★★	★	★	★	7
Choi JM (Biomed Chromatogr) 2016	GC	☆	☆	☆	★	★☆	★	★	★	5
Wang H (BMC Cancer) 2016	GC	☆	☆	☆	☆	★☆	★	★	★	4
Chan AW (Br J Cancer) 2016	GC	☆	☆	☆	★	★★	★	★	★	6
Kuligowski J (J Proteome Res) 2016	GC	☆	☆	☆	★	☆☆	★	★	★	4
Xu J (Sci Rep) 2016	EC	★	☆	☆	★	★★	★	★	★	7
Liang Q (Appl Biochem Biotechnol) 2015	GC	☆	☆	☆	★	★★	★	★	★	6
Mir SA (J Proteomics) 2015	EC	☆	☆	☆	★	★★	★	★	★	6
Jung J (Ann Surg Oncol) 2014	GC	☆	☆	☆	★	★★	★	★	★	6
Lo WY (Clin Chim Acta) 2014	GC	☆	☆	☆	★	★★	★	★	★	6

Table S2 Continued

Study	Group	Selection				Comparability	Exposure			Total
		Adequate definition of cases	Representativeness of the cases	Selection of controls	Definition of controls	Comparability of cases and controls*	Ascertainment of exposure	Same method of ascertainment for cases and controls	Non-response rate	
Chen JL (Electrophoresis) 2014	GC	☆	☆	☆	★	☆☆	★	★	★	4
Hur H (PLoS One) 2014	GC	☆	☆	☆	☆	★★	★	★	★	5
Yang T (Se Pu) 2014	GC	☆	☆	☆	★	☆☆	★	★	★	4
Kwon SY (Open Proteomics Journal) 2014	GC	☆	☆	☆	☆	★★	★	★	★	5
Yang Y (Anal Bioanal Chem) 2013	EC	☆	☆	☆	☆	★★	★	★	★	5
Zhang X (Biochim Biophys Acta) 2013	EC	☆	☆	☆	★	☆☆	★	★	★	4
Liu R (Int J Mol Sci) 2013	EC	☆	☆	☆	★	★★	★	★	★	6
Wang L (Molecular cancer) 2013	EC	☆	☆	☆	☆	★★	★	★	★	5
Song H (Chinese Journal of Clinical Nutrition) 2013	GC	☆	☆	☆	★	★★	★	★	★	6
Ikeda A (Biomed Chromatogr) 2012	GC and EC	☆	☆	☆	★	★☆	★	★	★	5
Song H (Braz J Med Biol Res) 2012	GC	☆	☆	☆	★	★★	★	★	★	6
Aa J (Metabolomics) 2012	GC	☆	☆	☆	★	★★	★	★	★	6
Hasim A (Mol Biol Rep) 2012	EC	☆	☆	☆	★	★★	★	★	★	6
Zhang J (PLoS One) 2012	EC	☆	☆	☆	★	☆☆	★	★	★	4
Davis VW (World J Surg Oncol) 2012	EC	☆	★	☆	★	★★	★	★	★	7
Song H (Chin Med J) 2012	GC	☆	☆	☆	☆	★★	★	★	★	5
Sun M (Chinese Journal of Gastroenterology) 2011	GC	☆	☆	☆	★	☆☆	★	★	★	4
Yu L (J Gastroenterol Hepatol) 2011	GC	☆	☆	☆	★	★☆	★	★	★	5
Song H (Oncol Rep) 2011	GC	☆	☆	☆	☆	★★	★	★	★	5
Zhang J (J Thorac Cardiovasc Surg) 2011	EC	★	☆	☆	★	☆☆	★	★	★	5
Wu H (Anal Bioanal Chem) 2010	GC	☆	☆	☆	☆	★★	★	★	★	5
Yakoub D (Cancer Res) 2010	EC	☆	☆	☆	★	★☆	★	★	★	5

Table S2 Continued

Study	Group	Selection				Comparability	Exposure			Total
		Adequate definition of cases	Representativeness of the cases	Selection of controls	Definition of controls	Comparability of cases and controls*	Ascertainment of exposure	Same method of ascertainment for cases and controls	Non-response rate	
Cai Z (Mol Cell Proteomics) 2010	GC	☆	☆	☆	☆	★★	★	★	★	5
Djukovic D (Rapid Commun Mass Spectrom) 2010	EC	☆	☆	☆	★	☆☆	★	★	★	4
Ayshamgul H (Chin J Oncol) 2010	EC	☆	☆	☆	★	★☆	★	★	★	5
Hirayama A (Cancer Res) 2009	GC	☆	☆	☆	☆	★★	★	★	★	5
Wu H (J Chromatogr B) 2009	EC	☆	☆	☆	☆	★★	★	★	★	5
Calabrese C (Cancer Epidemiol Biomarkers Prev) 2008	GC	☆	☆	☆	★	☆☆	★	★	★	4
Tugnoli V (Oncol Rep) 2006	GC	☆	☆	☆	★	☆☆	★	★	★	4
Mun CW (Magn Reson Imaging) 2004	GC	☆	☆	☆	☆	★★	★	★	★	5

*A maximum of 2 stars can be given in this category. GC, gastric cancer; EC, esophageal cancer.
